# Mahommedan Physicians

**Published:** 1846-04

**Authors:** 


*Mahommedan Physicians.*—A Bostonian, Thomas Wells, Esq., who has travelled extensively over the eastern part of the world, has written a book, entitled "Letters on Palestine." containing a vast variety of curious information, fitted to the benefit of all classes and ages of readers. On the 177th page, a notice of the medicine-taking propensity of the Sultan's obedient subjects, occurs, together with an illustration of the character and state of a hakim, or doctor.

"On our arrival at Zahle, we were beset with half the population of the place—some out of curiosity, some to get medicine, believing us to be doctors in the healing art. A late tourist travelling in this direction, gives an amusing account of a fat, jolly-looking dame, the picture of rude health, who insisted upon his feeling her pulse. It was in vain that he declared himself to be no hakim—physician. She would not be satisfied till he yielded to her request, and by assuring her that she stood in no need of medical aid. A dose of medicine would have been the most acceptable of all presents.

"Of the extreme eagerness of these people after physic, a ludicrous instance, related by the traveller just referred to, occurred at Constantinople. No person is allowed to practise there, as hakim, without a license from the Government, for which, of course, they are obliged to pay highly. A man had set up as doctor, without this diploma; the police were sent to apprehend him. Instead of seizing the culprit, they allowed him quietly to slip away, while they made a rush at his phials and gallipots, and swallowed, indiscriminately, the whole contents of his physic shop. Luckily, it consisted of simples only, and no harm was done.

"The practitioners in physic among the Mahommedans, are usually the barbers; in a country, of course, where every man's head is shaved, the professors of the healing art cannot fail to be numerous. Their knowledge of the science of medicine must necessarily be extremely confined. They perform a few surgical operations, says Dr. Hume, and are acquainted with the virtues of mercury and some standard medicines. The general remedy in cases of fever and other kinds of illness, is a saphie from a priest, which consists of some sentence from the Koran, written on a small piece of paper, and tied round the patient's neck."—*Bost. Med. and Surg. Journ.*

